# (*E*)-2-[4-(Dimethyl­amino)­styr­yl]-1-methyl­pyridinium 4-methyl­benzene­sulfonate monohydrate^1^

**DOI:** 10.1107/S1600536810026309

**Published:** 2010-07-10

**Authors:** Suchada Chantrapromma, Kullapa Chanawanno, Hoong-Kun Fun

**Affiliations:** a Crystal Materials Research Unit, Department of Chemistry, Faculty of Science, Prince of Songkla University, Hat-Yai, Songkhla 90112, Thailand; b X-ray Crystallography Unit, School of Physics, Universiti Sains Malaysia, 11800 USM, Penang, Malaysia

## Abstract

The cation of the title compound, C_16_H_19_N_2_^+^·C_7_H_7_O_3_S^−^·H_2_O, exists in the *E* configuration with respect to the C=C double bond and is essentially planar, the dihedral angle between the pyridinium and benzene rings being 3.55 (13)°. In the crystal, π-conjugated planes of cations and anions are inclined to each other at 84.30 (11)°. The crystal structure is stabilized by O—H⋯O hydrogen bonds and weak C—H⋯O inter­actions, which link the cations, anions and water mol­ecules into chains along the *b* axis. These chains are stacked along the *a* axis by π–π inter­actions with centroid–centroid distances of 3.6032 (16) and 3.6462 (16) Å.

## Related literature

For bond-length data, see Allen *et al.* (1987 ▶). For background to and applications of quarternary ammonium compounds and sulfonamides, see: Barlow *et al.* (1937 ▶); Ohkura *et al.* (2003 ▶); Pernak *et al.* (2001 ▶). For related structures, see: Chanawanno *et al.* (2010 ▶); Kobkeatthawin *et al.* (2009 ▶). For the stability of the temperature controller used in the data collection, see: Cosier & Glazer (1986 ▶).
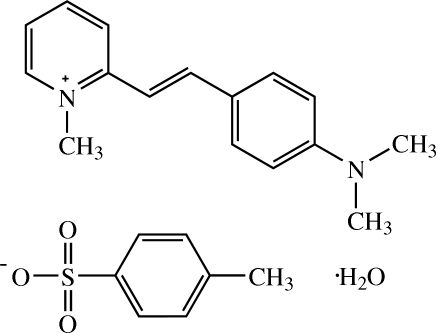


## Experimental

### 

#### Crystal data

C_16_H_19_N_2_^+^·C_7_H_7_O_3_S^−^·H_2_O
*M* *_r_* = 428.53
Triclinic, 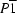

*a* = 7.3469 (9) Å
*b* = 9.8860 (12) Å
*c* = 15.5541 (19) Å
α = 75.801 (3)°
β = 79.438 (3)°
γ = 76.865 (2)°
*V* = 1056.8 (2) Å^3^
*Z* = 2
Mo *K*α radiation
μ = 0.19 mm^−1^
*T* = 100 K
0.47 × 0.13 × 0.06 mm

#### Data collection

Bruker APEXII DUO CCD area-detector diffractometer
Absorption correction: multi-scan (*SADABS*; Bruker, 2009 ▶) *T*_min_ = 0.919, *T*_max_ = 0.989
15705 measured reflections
4122 independent reflections
3307 reflections with *I* > 2σ(*I*)
*R*_int_ = 0.045

#### Refinement

*R*[*F* ^2^ > 2σ(*F* ^2^)] = 0.053
*wR*(*F* ^2^) = 0.156
*S* = 1.11
4122 reflections
282 parameters
H atoms treated by a mixture of independent and constrained refinement
Δρ_max_ = 0.51 e Å^−3^
Δρ_min_ = −0.56 e Å^−3^

### 

Data collection: *APEX2* (Bruker, 2009 ▶); cell refinement: *SAINT* (Bruker, 2009 ▶); data reduction: *SAINT*; program(s) used to solve structure: *SHELXTL* (Sheldrick, 2008 ▶); program(s) used to refine structure: *SHELXTL*; molecular graphics: *SHELXTL*; software used to prepare material for publication: *SHELXTL* and *PLATON* (Spek, 2009 ▶).

## Supplementary Material

Crystal structure: contains datablocks global, I. DOI: 10.1107/S1600536810026309/sj5034sup1.cif

Structure factors: contains datablocks I. DOI: 10.1107/S1600536810026309/sj5034Isup2.hkl

Additional supplementary materials:  crystallographic information; 3D view; checkCIF report

## Figures and Tables

**Table 1:** Hydrogen-bond geometry (Å, °)

*D*—H⋯*A*	*D*—H	H⋯*A*	*D*⋯*A*	*D*—H⋯*A*
O1*W*—H1*W*1⋯O2	0.83 (5)	1.96 (5)	2.774 (3)	170 (4)
O1*W*—H2*W*1⋯O1^i^	0.94 (4)	1.99 (4)	2.906 (3)	167 (3)
C1—H1*A*⋯O1^ii^	0.93	2.55	3.424 (3)	157
C2—H2*A*⋯O1^iii^	0.93	2.54	3.382 (4)	150
C4—H4*A*⋯O1*W*	0.93	2.35	3.222 (4)	157
C6—H6*A*⋯O3^iv^	0.93	2.53	3.456 (4)	176
C9—H9*A*⋯O2	0.93	2.49	3.376 (3)	158
C13—H13*A*⋯O3^iv^	0.93	2.49	3.390 (4)	164
C14—H14*B*⋯O1^ii^	0.96	2.56	3.479 (4)	161

Symmetry codes: (i) 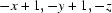; (ii) 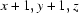; (iii) 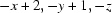; (iv) 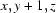.

**Fig. 1.**
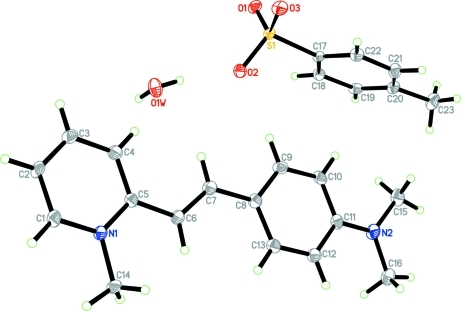
The asymmetric unit of (I) showing 50% probability displacement ellipsoids and the atom-numbering scheme.

**Fig. 2.**
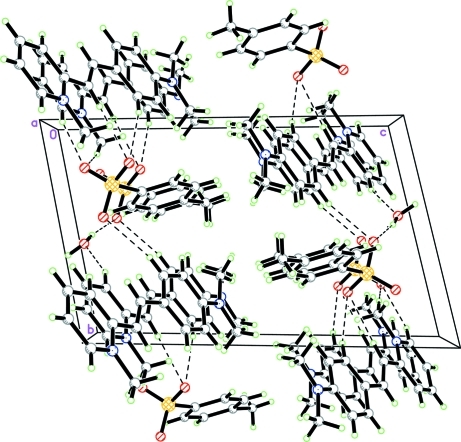
The crystal packing of (I) viewed along the a axis. The O—H···O hydrogen bonds and weak C—H···O interactions are drawn as dashed lines.

## supplementary crystallographic information

### Comment

Quarternary ammonium compounds and sulfonamide drugs are the interesting antibacterial agents. They are widely used in industrial disinfection and hospital treatment (Barlow *et al.*, 1937; Ohkura *et al.*, 2003). Pyridinium derivatives  represent a class of synthetic quarternary ammonium compounds that show significant antibacterial activity. (Pernak *et al.*, 2001). The title compound was synthesized based on the combination of pyridinium and sulfonamide chemophores in order to yield a potent disinfectant. Our biological actvity results showed that the title compound was moderately active against Gram-positive bacteria *ie* Methicillin-Resistant *Staphylococcus aureus* with the MIC = 37.5 µg/ml. However it was inactive against the Gram-negative bacteria we tested which are *Pseudomonas aeruginosa*, *Salmonella typhi* and *Shigella sonnei* (Chanawanno *et al.*, 2010). Herein its crystal structure is reported.

Fig. 1 shows the asymmetric unit of the title compound (I) which consists of the C_16_H_19_N_2_^+^ cation, C_7_H_7_O_3_S^-^ anion and one H_2_O molecule. The cation exists in the *E* configuration with respect to the C6═C7 double bond [1.343 (4) Å]. The cation is essentially planar with the dihedral angle between the pyridinium and the dimethylaminophenyl rings being 3.55 (13)° and with the torsion angle C5–C6–C7–C8 = 176.3 (3)°. Both methyl groups of dimethylamino moiety are slightly twisted from the mean plane of the attached C8–C13 ring as indicated by the torsion angles C15—N2–C11–C10 = 9.3 (4)° and C16–N2–C11–C12 = 3.5 (4)°. The relative arrangement of cation and anion is shown by the interplanar angle between the mean plane of the π-conjugate system (C1–C13/N1) of the cation and the C17–C22 benzene ring of the anion being 84.30 (11)°. The bond lengths (Allen *et al.*, 1987) and angles in (I) are in normal ranges and comparable with a related structure (Kobkeatthawin *et al.*, 2009).

In the crystal packing, all O atoms of the sulfonate group are involved in weak C—H···O interactions (Table 1). The cation is linked to both the anion and water molecule by weak C—H···O interactions, and the anion is linked to the water molecule by O—H···O hydrogen bond. These three molecules are linked into chains along the *b* axis (Table 1, Fig. 2). These chains are stacked along the *a* axis (Fig. 2) by π–π interactions with the distances *Cg*_1_···*Cg*_1_ = 3.6032 (16) Å (symmetry code: 2 - *x*, 2 - *y*, -*z*) and *Cg*_1_···*Cg*_2_ = 3.6462 (16) Å (symmetry code: 1 - *x*, *y*, *z*); *Cg*_1_ and *Cg*_2_ are the centroids of the N1/C1–C5 and C8–C13 rings, respectively.

### Experimental

The title compound was prepared by the reported procedure (Chanawanno *et al.*, 2010). Orange needle-shaped single crystals of the title compound suitable for *x*-ray structure determination were recrystalized from methanol by slow evaporation of the solvent at room temperature after a few weeks. Mp. 468–469 K.

### Refinement

Water H atoms were located in difference maps and refined isotropically. The remaining H atoms were placed in calculated positions with d(C—H) = 0.93 Å, *U*_iso_=1.2*U*_eq_(C) for aromatic and CH and 0.96 Å, *U*_iso_ = 1.5*U*_eq_(C) for CH_3_ atoms. A rotating group model was used for the methyl groups. The highest residual electron density peak is located at 1.08 Å from O1 and the deepest hole is located at 0.85 Å from S1.

### Crystal data

**Table:**

C_16_H_19_N_2_^+^·C_7_H_7_O_3_S^−^·H_2_O	*Z* = 2
*M**_r_* = 428.53	*F*(000) = 456
Triclinic, *P*1	*D*_x_ = 1.347 Mg m^−^^3^
Hall symbol: -P 1	Melting point = 468–469 K
*a* = 7.3469 (9) Å	Mo *K*α radiation, λ = 0.71073 Å
*b* = 9.8860 (12) Å	Cell parameters from 4122 reflections
*c* = 15.5541 (19) Å	θ = 1.4–26.0°
α = 75.801 (3)°	µ = 0.19 mm^−^^1^
β = 79.438 (3)°	*T* = 100 K
γ = 76.865 (2)°	Needle, orange
*V* = 1056.8 (2) Å^3^	0.47 × 0.13 × 0.06 mm

### Data collection

**Table:**

Bruker APEXII DUO CCD area-detector diffractometer	4122 independent reflections
Radiation source: sealed tube	3307 reflections with *I* > 2σ(*I*)
graphite	*R*_int_ = 0.045
φ and ω scans	θ_max_ = 26.0°, θ_min_ = 1.4°
Absorption correction: multi-scan (*SADABS*; Bruker, 2009)	*h* = −9→9
*T*_min_ = 0.919, *T*_max_ = 0.989	*k* = −12→12
15705 measured reflections	*l* = −19→19

### Refinement

**Table:**

Refinement on *F*^2^	Primary atom site location: structure-invariant direct methods
Least-squares matrix: full	Secondary atom site location: difference Fourier map
*R*[*F*^2^ > 2σ(*F*^2^)] = 0.053	Hydrogen site location: inferred from neighbouring sites
*wR*(*F*^2^) = 0.156	H atoms treated by a mixture of independent and constrained refinement
*S* = 1.11	*w* = 1/[σ^2^(*F*_o_^2^) + (0.069*P*)^2^ + 1.5338*P*] where *P* = (*F*_o_^2^ + 2*F*_c_^2^)/3
4122 reflections	(Δ/σ)_max_ = 0.001
282 parameters	Δρ_max_ = 0.51 e Å^−^^3^
0 restraints	Δρ_min_ = −0.56 e Å^−^^3^

### Special details

**Table:**

Experimental. The crystal was placed in the cold stream of an Oxford Cryosystems Cobra open-flow nitrogen cryostat (Cosier & Glazer, 1986) operating at 100.0 (1) K.
Geometry. All e.s.d.'s (except the e.s.d. in the dihedral angle between two l.s. planes) are estimated using the full covariance matrix. The cell e.s.d.'s are taken into account individually in the estimation of e.s.d.'s in distances, angles and torsion angles; correlations between e.s.d.'s in cell parameters are only used when they are defined by crystal symmetry. An approximate (isotropic) treatment of cell e.s.d.'s is used for estimating e.s.d.'s involving l.s. planes.
Refinement. Refinement of *F*^2^ against ALL reflections. The weighted *R*-factor *wR* and goodness of fit *S* are based on *F*^2^, conventional *R*-factors *R* are based on *F*, with *F* set to zero for negative *F*^2^. The threshold expression of *F*^2^ > σ(*F*^2^) is used only for calculating *R*-factors(gt) *etc*. and is not relevant to the choice of reflections for refinement. *R*-factors based on *F*^2^ are statistically about twice as large as those based on *F*, and *R*- factors based on ALL data will be even larger.

### Fractional atomic coordinates and isotropic or equivalent isotropic displacement parameters (Å^2^)

**Table:**

	*x*	*y*	*z*	*U*_iso_*/*U*_eq_	
N1	1.0171 (3)	0.9737 (2)	0.12371 (15)	0.0145 (5)	
N2	−0.1299 (3)	0.8266 (3)	0.39380 (16)	0.0215 (5)	
C1	1.1969 (4)	0.9572 (3)	0.08076 (18)	0.0176 (6)	
H1A	1.2683	1.0256	0.0766	0.021*	
C2	1.2745 (4)	0.8414 (3)	0.04353 (19)	0.0198 (6)	
H2A	1.3976	0.8309	0.0143	0.024*	
C3	1.1662 (4)	0.7399 (3)	0.05011 (18)	0.0189 (6)	
H3A	1.2161	0.6606	0.0252	0.023*	
C4	0.9842 (4)	0.7579 (3)	0.09382 (18)	0.0170 (6)	
H4A	0.9115	0.6905	0.0975	0.020*	
C5	0.9067 (4)	0.8750 (3)	0.13271 (18)	0.0152 (5)	
C6	0.7180 (4)	0.8965 (3)	0.18253 (18)	0.0166 (6)	
H6A	0.6710	0.9821	0.2007	0.020*	
C7	0.6089 (4)	0.7978 (3)	0.20334 (18)	0.0177 (6)	
H7A	0.6566	0.7156	0.1812	0.021*	
C8	0.4235 (4)	0.8062 (3)	0.25704 (18)	0.0166 (5)	
C9	0.3246 (4)	0.6955 (3)	0.26819 (19)	0.0182 (6)	
H9A	0.3816	0.6174	0.2431	0.022*	
C10	0.1460 (4)	0.6991 (3)	0.31509 (18)	0.0168 (6)	
H10A	0.0860	0.6230	0.3223	0.020*	
C11	0.0532 (4)	0.8173 (3)	0.35233 (18)	0.0163 (5)	
C12	0.1546 (4)	0.9257 (3)	0.34420 (18)	0.0176 (6)	
H12A	0.0992	1.0026	0.3707	0.021*	
C13	0.3347 (4)	0.9203 (3)	0.29778 (18)	0.0172 (6)	
H13A	0.3980	0.9937	0.2935	0.021*	
C14	0.9449 (4)	1.1036 (3)	0.16027 (19)	0.0186 (6)	
H14A	0.8340	1.1563	0.1345	0.028*	
H14B	1.0398	1.1612	0.1460	0.028*	
H14C	0.9142	1.0774	0.2241	0.028*	
C15	−0.2224 (4)	0.7057 (3)	0.4121 (2)	0.0236 (6)	
H15A	−0.2238	0.6787	0.3570	0.035*	
H15B	−0.3496	0.7309	0.4400	0.035*	
H15C	−0.1552	0.6275	0.4515	0.035*	
C16	−0.2284 (4)	0.9532 (3)	0.4263 (2)	0.0248 (6)	
H16A	−0.2241	1.0353	0.3784	0.037*	
H16B	−0.1688	0.9626	0.4740	0.037*	
H16C	−0.3574	0.9455	0.4481	0.037*	
S1	0.39079 (9)	0.30482 (7)	0.18364 (5)	0.01550 (19)	
O1	0.3624 (3)	0.2330 (2)	0.11664 (13)	0.0202 (4)	
O2	0.4315 (3)	0.4458 (2)	0.14332 (13)	0.0214 (5)	
O3	0.5235 (3)	0.2178 (2)	0.24342 (14)	0.0205 (4)	
C17	0.1685 (3)	0.3327 (3)	0.25164 (18)	0.0138 (5)	
C18	0.0064 (4)	0.3932 (3)	0.21068 (18)	0.0157 (5)	
H18A	0.0153	0.4239	0.1488	0.019*	
C19	−0.1682 (4)	0.4069 (3)	0.26349 (19)	0.0170 (6)	
H19A	−0.2767	0.4449	0.2364	0.020*	
C20	−0.1835 (4)	0.3651 (3)	0.35578 (19)	0.0182 (6)	
C21	−0.0197 (4)	0.3085 (3)	0.39579 (19)	0.0196 (6)	
H21A	−0.0280	0.2819	0.4578	0.024*	
C22	0.1561 (4)	0.2916 (3)	0.34368 (18)	0.0166 (6)	
H22A	0.2647	0.2529	0.3707	0.020*	
C23	−0.3743 (4)	0.3757 (3)	0.4126 (2)	0.0266 (7)	
H23A	−0.3581	0.3418	0.4746	0.040*	
H23B	−0.4472	0.3190	0.3963	0.040*	
H23C	−0.4389	0.4730	0.4031	0.040*	
O1W	0.7344 (3)	0.5643 (2)	0.04598 (15)	0.0251 (5)	
H1W1	0.645 (7)	0.524 (5)	0.070 (3)	0.056 (13)*	
H2W1	0.699 (5)	0.618 (4)	−0.009 (3)	0.033 (10)*	

### Atomic displacement parameters (Å^2^)

**Table:**

	*U*^11^	*U*^22^	*U*^33^	*U*^12^	*U*^13^	*U*^23^
N1	0.0088 (10)	0.0153 (11)	0.0197 (11)	−0.0024 (9)	−0.0005 (9)	−0.0051 (9)
N2	0.0114 (12)	0.0250 (13)	0.0286 (13)	−0.0059 (10)	0.0045 (10)	−0.0096 (10)
C1	0.0078 (12)	0.0207 (14)	0.0230 (14)	−0.0019 (10)	−0.0013 (10)	−0.0038 (11)
C2	0.0083 (13)	0.0234 (14)	0.0237 (14)	0.0021 (11)	0.0016 (11)	−0.0049 (11)
C3	0.0164 (14)	0.0163 (13)	0.0209 (14)	0.0024 (11)	−0.0021 (11)	−0.0034 (11)
C4	0.0140 (13)	0.0148 (13)	0.0213 (14)	−0.0014 (10)	−0.0025 (11)	−0.0032 (11)
C5	0.0115 (13)	0.0154 (13)	0.0180 (13)	−0.0019 (10)	−0.0030 (10)	−0.0019 (10)
C6	0.0109 (13)	0.0168 (13)	0.0203 (14)	−0.0003 (10)	−0.0005 (10)	−0.0040 (11)
C7	0.0113 (13)	0.0174 (13)	0.0231 (14)	−0.0003 (10)	−0.0018 (11)	−0.0046 (11)
C8	0.0105 (13)	0.0167 (13)	0.0211 (14)	−0.0016 (10)	−0.0023 (10)	−0.0022 (10)
C9	0.0136 (14)	0.0154 (13)	0.0251 (14)	−0.0002 (11)	−0.0034 (11)	−0.0050 (11)
C10	0.0129 (13)	0.0149 (13)	0.0239 (14)	−0.0069 (10)	−0.0024 (11)	−0.0026 (11)
C11	0.0102 (13)	0.0192 (13)	0.0179 (13)	−0.0028 (10)	−0.0011 (10)	−0.0018 (10)
C12	0.0149 (13)	0.0166 (13)	0.0213 (14)	−0.0028 (11)	−0.0015 (11)	−0.0052 (11)
C13	0.0117 (13)	0.0175 (13)	0.0221 (14)	−0.0035 (10)	−0.0024 (11)	−0.0028 (11)
C14	0.0114 (13)	0.0176 (13)	0.0275 (15)	−0.0020 (10)	0.0016 (11)	−0.0100 (11)
C15	0.0147 (14)	0.0294 (16)	0.0268 (15)	−0.0090 (12)	0.0022 (12)	−0.0054 (12)
C16	0.0145 (14)	0.0289 (16)	0.0283 (16)	−0.0032 (12)	0.0053 (12)	−0.0082 (13)
S1	0.0063 (3)	0.0145 (3)	0.0248 (4)	−0.0030 (2)	0.0034 (2)	−0.0058 (3)
O1	0.0130 (10)	0.0195 (10)	0.0287 (11)	−0.0047 (8)	0.0046 (8)	−0.0104 (8)
O2	0.0116 (10)	0.0181 (10)	0.0320 (11)	−0.0049 (8)	0.0060 (8)	−0.0052 (8)
O3	0.0055 (9)	0.0211 (10)	0.0332 (11)	0.0004 (7)	−0.0020 (8)	−0.0057 (8)
C17	0.0055 (12)	0.0123 (12)	0.0236 (14)	−0.0031 (9)	0.0029 (10)	−0.0064 (10)
C18	0.0112 (13)	0.0150 (13)	0.0210 (14)	−0.0029 (10)	−0.0015 (10)	−0.0040 (10)
C19	0.0086 (12)	0.0130 (13)	0.0296 (15)	0.0001 (10)	−0.0039 (11)	−0.0059 (11)
C20	0.0087 (13)	0.0195 (13)	0.0269 (15)	−0.0050 (10)	0.0044 (11)	−0.0086 (11)
C21	0.0153 (14)	0.0233 (14)	0.0196 (14)	−0.0052 (11)	0.0006 (11)	−0.0043 (11)
C22	0.0091 (13)	0.0171 (13)	0.0244 (14)	−0.0032 (10)	−0.0028 (10)	−0.0049 (11)
C23	0.0117 (14)	0.0357 (17)	0.0310 (16)	−0.0056 (12)	0.0055 (12)	−0.0098 (13)
O1W	0.0169 (11)	0.0311 (12)	0.0287 (12)	−0.0126 (9)	0.0002 (9)	−0.0038 (10)

### Geometric parameters (Å, °)

**Table:**

N1—C1	1.360 (3)	C14—H14A	0.9600
N1—C5	1.372 (3)	C14—H14B	0.9600
N1—C14	1.480 (3)	C14—H14C	0.9600
N2—C11	1.374 (3)	C15—H15A	0.9600
N2—C15	1.450 (4)	C15—H15B	0.9600
N2—C16	1.454 (4)	C15—H15C	0.9600
C1—C2	1.371 (4)	C16—H16A	0.9600
C1—H1A	0.9300	C16—H16B	0.9600
C2—C3	1.390 (4)	C16—H16C	0.9600
C2—H2A	0.9300	S1—O3	1.454 (2)
C3—C4	1.378 (4)	S1—O2	1.4553 (19)
C3—H3A	0.9300	S1—O1	1.464 (2)
C4—C5	1.396 (4)	S1—C17	1.780 (3)
C4—H4A	0.9300	C17—C22	1.381 (4)
C5—C6	1.456 (4)	C17—C18	1.396 (4)
C6—C7	1.343 (4)	C18—C19	1.389 (4)
C6—H6A	0.9300	C18—H18A	0.9300
C7—C8	1.458 (4)	C19—C20	1.384 (4)
C7—H7A	0.9300	C19—H19A	0.9300
C8—C13	1.402 (4)	C20—C21	1.395 (4)
C8—C9	1.406 (4)	C20—C23	1.511 (4)
C9—C10	1.377 (4)	C21—C22	1.392 (4)
C9—H9A	0.9300	C21—H21A	0.9300
C10—C11	1.411 (4)	C22—H22A	0.9300
C10—H10A	0.9300	C23—H23A	0.9600
C11—C12	1.407 (4)	C23—H23B	0.9600
C12—C13	1.383 (4)	C23—H23C	0.9600
C12—H12A	0.9300	O1W—H1W1	0.83 (5)
C13—H13A	0.9300	O1W—H2W1	0.94 (4)
			
C1—N1—C5	121.8 (2)	H14A—C14—H14B	109.5
C1—N1—C14	117.0 (2)	N1—C14—H14C	109.5
C5—N1—C14	121.2 (2)	H14A—C14—H14C	109.5
C11—N2—C15	120.3 (2)	H14B—C14—H14C	109.5
C11—N2—C16	120.6 (2)	N2—C15—H15A	109.5
C15—N2—C16	118.9 (2)	N2—C15—H15B	109.5
N1—C1—C2	121.1 (2)	H15A—C15—H15B	109.5
N1—C1—H1A	119.5	N2—C15—H15C	109.5
C2—C1—H1A	119.5	H15A—C15—H15C	109.5
C1—C2—C3	118.9 (2)	H15B—C15—H15C	109.5
C1—C2—H2A	120.5	N2—C16—H16A	109.5
C3—C2—H2A	120.5	N2—C16—H16B	109.5
C4—C3—C2	119.4 (2)	H16A—C16—H16B	109.5
C4—C3—H3A	120.3	N2—C16—H16C	109.5
C2—C3—H3A	120.3	H16A—C16—H16C	109.5
C3—C4—C5	121.5 (2)	H16B—C16—H16C	109.5
C3—C4—H4A	119.3	O3—S1—O2	113.73 (11)
C5—C4—H4A	119.3	O3—S1—O1	113.30 (12)
N1—C5—C4	117.3 (2)	O2—S1—O1	111.86 (12)
N1—C5—C6	118.9 (2)	O3—S1—C17	106.05 (12)
C4—C5—C6	123.8 (2)	O2—S1—C17	105.69 (11)
C7—C6—C5	122.8 (2)	O1—S1—C17	105.35 (11)
C7—C6—H6A	118.6	C22—C17—C18	120.5 (2)
C5—C6—H6A	118.6	C22—C17—S1	120.3 (2)
C6—C7—C8	126.8 (3)	C18—C17—S1	119.2 (2)
C6—C7—H7A	116.6	C19—C18—C17	119.1 (2)
C8—C7—H7A	116.6	C19—C18—H18A	120.4
C13—C8—C9	117.2 (2)	C17—C18—H18A	120.4
C13—C8—C7	123.7 (2)	C20—C19—C18	121.2 (2)
C9—C8—C7	119.0 (2)	C20—C19—H19A	119.4
C10—C9—C8	122.1 (2)	C18—C19—H19A	119.4
C10—C9—H9A	119.0	C19—C20—C21	118.9 (2)
C8—C9—H9A	119.0	C19—C20—C23	120.9 (3)
C9—C10—C11	120.6 (2)	C21—C20—C23	120.2 (3)
C9—C10—H10A	119.7	C22—C21—C20	120.6 (3)
C11—C10—H10A	119.7	C22—C21—H21A	119.7
N2—C11—C12	121.5 (2)	C20—C21—H21A	119.7
N2—C11—C10	121.1 (2)	C17—C22—C21	119.6 (2)
C12—C11—C10	117.4 (2)	C17—C22—H22A	120.2
C13—C12—C11	121.5 (2)	C21—C22—H22A	120.2
C13—C12—H12A	119.3	C20—C23—H23A	109.5
C11—C12—H12A	119.3	C20—C23—H23B	109.5
C12—C13—C8	121.1 (2)	H23A—C23—H23B	109.5
C12—C13—H13A	119.5	C20—C23—H23C	109.5
C8—C13—H13A	119.5	H23A—C23—H23C	109.5
N1—C14—H14A	109.5	H23B—C23—H23C	109.5
N1—C14—H14B	109.5	H1W1—O1W—H2W1	105 (4)
			
C5—N1—C1—C2	0.8 (4)	C9—C10—C11—N2	175.4 (3)
C14—N1—C1—C2	−178.9 (3)	C9—C10—C11—C12	−3.8 (4)
N1—C1—C2—C3	0.1 (4)	N2—C11—C12—C13	−176.0 (3)
C1—C2—C3—C4	−0.1 (4)	C10—C11—C12—C13	3.1 (4)
C2—C3—C4—C5	−0.8 (4)	C11—C12—C13—C8	−0.1 (4)
C1—N1—C5—C4	−1.7 (4)	C9—C8—C13—C12	−2.3 (4)
C14—N1—C5—C4	178.1 (2)	C7—C8—C13—C12	176.7 (3)
C1—N1—C5—C6	177.6 (2)	O3—S1—C17—C22	8.8 (2)
C14—N1—C5—C6	−2.6 (4)	O2—S1—C17—C22	−112.3 (2)
C3—C4—C5—N1	1.7 (4)	O1—S1—C17—C22	129.2 (2)
C3—C4—C5—C6	−177.6 (3)	O3—S1—C17—C18	−169.54 (19)
N1—C5—C6—C7	−172.2 (3)	O2—S1—C17—C18	69.4 (2)
C4—C5—C6—C7	7.1 (4)	O1—S1—C17—C18	−49.1 (2)
C5—C6—C7—C8	176.3 (3)	C22—C17—C18—C19	−2.3 (4)
C6—C7—C8—C13	−2.3 (4)	S1—C17—C18—C19	176.08 (19)
C6—C7—C8—C9	176.7 (3)	C17—C18—C19—C20	1.6 (4)
C13—C8—C9—C10	1.6 (4)	C18—C19—C20—C21	0.1 (4)
C7—C8—C9—C10	−177.4 (3)	C18—C19—C20—C23	−177.9 (2)
C8—C9—C10—C11	1.5 (4)	C19—C20—C21—C22	−1.3 (4)
C15—N2—C11—C12	−171.6 (3)	C23—C20—C21—C22	176.7 (2)
C16—N2—C11—C12	3.5 (4)	C18—C17—C22—C21	1.1 (4)
C15—N2—C11—C10	9.3 (4)	S1—C17—C22—C21	−177.2 (2)
C16—N2—C11—C10	−175.6 (3)	C20—C21—C22—C17	0.7 (4)

### Hydrogen-bond geometry (Å, °)

**Table:**

*D*—H···*A*	*D*—H	H···*A*	*D*···*A*	*D*—H···*A*
O1W—H1W1···O2	0.83 (5)	1.96 (5)	2.774 (3)	170 (4)
O1W—H2W1···O1^i^	0.94 (4)	1.99 (4)	2.906 (3)	167 (3)
C1—H1A···O1^ii^	0.93	2.55	3.424 (3)	157
C2—H2A···O1^iii^	0.93	2.54	3.382 (4)	150
C4—H4A···O1W	0.93	2.35	3.222 (4)	157
C6—H6A···O3^iv^	0.93	2.53	3.456 (4)	176
C9—H9A···O2	0.93	2.49	3.376 (3)	158
C13—H13A···O3^iv^	0.93	2.49	3.390 (4)	164
C14—H14B···O1^ii^	0.96	2.56	3.479 (4)	161
C22—H22A···O3	0.93	2.51	2.894 (3)	105

Symmetry codes:  (i) −*x*+1, −*y*+1, −*z*; (ii) *x*+1, *y*+1, *z*; (iii) −*x*+2, −*y*+1, −*z*; (iv) *x*, *y*+1, *z*.
